# Overexpression of S100A7 Protects LPS-Induced Mitochondrial Dysfunction and Stimulates IL-6 and IL-8 in HaCaT Cells

**DOI:** 10.1371/journal.pone.0092927

**Published:** 2014-03-26

**Authors:** Wenyan Sun, Yan Zheng, Zhuoyang Lu, Yang Cui, Qiong Tian, Shengxiang Xiao, Feng Liu, Jiankang Liu

**Affiliations:** 1 Center for Mitochondrial Biology and Medicine, The Key Laboratory of Biomedical Information Engineering of Ministry of Education, School of Life Science and Technology and Frontier Institute of Science and Technology, Xi’an Jiaotong University, Xi’an, P. R. China; 2 Department of Dermatology, the 2nd Affiliated Hospital of Xi’an Jiaotong University, Xi’an, P. R. China; 3 Department of Medicine, University of California Irvine Medical School, Irvine, United States of America; 4 Chao Family Comprehensive Cancer Center, University of California Irvine Medical School, Irvine, United States of America; 5 Department of Pharmacology and Pharmaceutical Sciences, School of Pharmacy, University of Southern California, Los Angeles, United States of America; University of Tennessee, United States of America

## Abstract

**Background:**

S100A7 (or psoriasin) is distributed in the cytoplasm of keratinocytes of normal human epidermis, and it is overexpressed in many epidermal inflammatory diseases. Lipopolysaccharide (LPS) induces mitochondrial function changes, which play important roles in multiple cellular mechanisms including inflammation. Although S100A7 expression is regulated by various factors in the human epidermis during inflammation, whether S100A7 interacts with mitochondria in keratinocytes is not clear.

**Objectives:**

Our study was designed to investigate whether S100A7 could prohibit mitochondrial dysfunction and stimulate cytokines in cultured normal HaCaT cells treated with LPS.

**Results:**

We generated HaCaT cells that constitutively express enhanced green fluorescence protein (EGFP)-S100A7 (S100A7-EGFP) or EGFP alone, as a control. Here, we show that S100A7-EGFP HaCaT cells exhibit an increase in mitochondrial DNA (mtDNA) copy number and mitochondrial membrane potential (MMP). qRT-PCR revealed that expression of three main mitochondrial biogenesis-associated genes was significantly increased: PPAR-coactivator-1alpha (PGC-1α), the mitochondrial transcription factor A (Tfam) and nuclear respiratory factor-1 (NRF1). S100A7 overexpression increased mtDNA content and effectively increased intracellular adenosine 5′-triphosphate (ATP) production, while decreasing reactive oxygen species (ROS) generation. S100A7 overexpression also significantly decreased the expression of Mfn2 and increased DRP1 expression compared with control EGFP cells. S100A7 down-regulated the expression of the autophagy-related proteins Beclin-1 and LC3B. S100A7 also increased expression of IL-6 and IL-8 cytokines. Knockdown of S100A7 decreased MMP and disrupted mitochondrial homeostasis.

**Conclusions:**

These findings demonstrate that S100A7 stimulates mitochondrial biogenesis and increases mitochondrial function in HaCaT cells treated with LPS; and S100A7 also promotes secretion of IL-6 and IL-8.

## Introduction

S100A7 (psoriasin), a member of the S100 family of EF-hand calcium-binding proteins, is distributed in the cytoplasm of keratinocytes of normal human epidermis, is present at the cell cytoplasm in terminally differentiated keratinocytes [1] and is expressed in mammary epithelial cells [2]. S100A7 is encoded in the epidermal differentiation complex on chromosome 1q21 [3], hence it may play an important role in epidermal biology. S100A7 binds calcium, and its basal expression is influenced by extracellular calcium levels [4], [5]. In addition S100A7 expression is regulated by various agents including all-trans retinoic acid, retinoid and UV light [6]. Cytokines including oncostatin M, interleukin (IL)-6 and IL-1 induced S100A7 expression directly or indirectly through STAT3 pathways [7]–[9].

S100A7 is overexpressed in many epidermal inflammatory diseases, including atopic dermatitis, mycosis fungoides, Darier’s disease, and inflammatory lichen sclerosus and atrophicus [10], suggesting that S100A7 may play a role in the inflammatory process. It was also up-regulated in invasive skin cancers [11] and in a subset of in situ and invasive breast carcinomas [12].

Mitochondria play key roles in multiple cellular mechanisms, such as intracellular adenosine 5′-triphosphate (ATP) biosynthesis through oxidative phosphorylation, cell death regulation and reactive oxygen species (ROS) production. ROS are important for amplifying pro-inflammatory pathways, such as NF-κB and JNK. Early ROS production, after IL-1β and lipopolysaccharide (LPS) stimulation, is a key messenger for subsequent NF-κB activation [13].

Mitophagy, the selective degradation of mitochondria [14], plays a crucial role in maintaining mitochondrial homeostasis. The accumulation of damaged mitochondria is a major cause of inflammation. Stimulation of autophagy may mediate cytoprotective and anti-inflammatory effects that can at least partially be ascribed to the removal of dysfunctional mitochondria [15]. However, to the best of our knowledge, how S100A7 affects mitochondrial biogenesis and function in LPS-induced HaCaT cells is largely unexplored. Therefore, our study was designed to investigate whether S100A7 could prohibit mitochondrial dysfunction and stimulate cytokines in cultured normal human keratinocytes treated with LPS.

## Materials and Methods

### Antibodies and Reagents

All chemicals used were analytical, high- purity reagents from Sigma–Aldrich (St Louis, MO) or local vendors.

### Cell Culture and Stable Cell Lines

The normal human HaCaT cell line was purchased from the Chinese Academy of Sciences (Kunming, China) and cultured in DMEM supplemented with 10% fetal calf serum. The complete S100A7 region was amplified by reverse transcription PCR from HaCaT cells using the following primers: sense 5′-GGCAAGCTTAAAGCAAAGATGAGCAACAC-3′; antisense 5′-TATGGATCCTGGGGTCTCTGGAG-3′. The resulting amplicon was then inserted between the BamHI and Hind III sites of pEGFP-C3 (Clontech, Hampshire, UK) to create the S100A7-EGFP construct. The plasmid was verified by sequence analysis to confirm the absence of mutations. The stable S100A7-EGFP and the vehicle-EGFP cell lines were constructed as previously described [16]. The transfected HaCaT cells were subcultured in the presence of 600 ng ml^–1^ of G418 (Invitrogen, Carlsbad, CA).

### Cell Fixation and Immunofluorescence

S100A7-EGFP and vehicle-EGFP cells were cultured on coverslips, then fixed with 4% paraformaldehyde (Sigma-Aldrich) for 15 min and permeabilized with 0.1% Triton X-100/PBS. After blocking in 5% BSA/PBS, the cells were incubated with a primary antibody for S100A7 for 3 h, washed and then incubated with the appropriately conjugated secondary antibody (Beyotime, Jiangsu, China) for 60 min. Nuclei were stained with 4,6-diamidino-2-phenylindole (DAPI; Sigma). Immunofluorescence was visualized using a confocal laser scanning microscope (Zeiss, Oberkochen, Germany).

### Assay for Mitochondrial Membrane Potential (MMP)

The MMP was assessed as previously described [17]. Cells were incubated with 5 μmol/L 5,5′,6,6′-tetrachloro-1,1′,3,3′- tetraethylbenzimidazolylcarbocyanine iodide (JC-1) (Invitrogen) at 37°C for 60 min. The fluorescence labeled cells were washed with PBS and analyzed by a BD FACSCalibur Flow Cytometry System (excitation: 485 nm; emission: 530 nm, 590 nm) (BD Biosciences, San Diego, CA). The ratio of fluorescence at 590 nm versus 530 nm emission was used for quantitating the MMP.

### Assay for Intracellular ATP Levels

ATP concentrations were assayed as previously described [18]. Briefly, cells were lysed and then centrifuged at approximately 15,000 g for 10 min at 4°C. The supernatants were removed and transferred to ATP assay mix working solution (Sigma), and the amount of light emitted was measured with a luminometer (Thermo Scientific Luminoskan Ascent, Waltham, MA) immediately. The luminescence data were normalized by sample protein amounts.

### Intracellular ROS Assay

ROS generation was detected using dichlorodihydrofluorescein diacetate (H2DCF-DA) [19]. Fluorescence was determined with a fluorescence spectrometer (Flex StationII 384, Molecular Devices) at 485 nm (excitation) and 538 nm (emission). Cellular oxidant levels were expressed as relative DCF fluorescence per sample protein amounts (measured using a BCA assay).

### Mitochondrial DNA (mtDNA) Quantification

The mtDNA content was determined as previously described [18].

### RNA Isolation and qRT-PCR

Total RNA was extracted using the TRIzol reagent (Invitrogen). 1.5 μg of RNA was reverse transcribed, and the synthesized cDNA was amplified in triplicate using specific primers (IL-6: sense:5′-AAGCCAGAGCTGTGCAGATGAGTA-3′; antisense: 5′-TGTCCTGCAGCCACTGGTTC-3′; IL-8: sense: 5′-GTCCTTGTTCCACTGTGCCT-3′; antisense: 5′-GCTTCCACATGTCCTCACAA-3′; TNF-α: sense: 5′-TCCTTCAGACACCCTCAACC-3′; antisense: 5′-AGGCCCCAGTTTGAATTCTT-3′; IL-1α: sense: 5′-TGGCTCATTTTCCCTCAAAAGTTG-3′; antisense: 5′-AGAAATCGTGAAATCCGAAGTCAAG-3′; IL-1β: sense: 5′-CCAGGGACAGGATATGGAGCA-3′; antisense: 5′-TTCAACACGCAGGACAGGTACAG-3′.). The nuclear respiratory factor-1 (NRF1), transcription factor A (Tfam) and PPAR-coactivator-1alpha (PGC-1α) primers were previously described [20]. Quantitative PCR was performed using a real-time PCR system (Eppendorf, Germany). Reactions were performed as previously described [21].

### ELISA for Cytokines

The TNF-α, IL-6, IL-8, IL-1α and IL-1β levels in the cell culture supernatants were measured by ELISA using kits purchased from Excell Biology, Inc. (Shanghai, China). The optical density of each well was determined using a microplate reader (Bio-Rad, Hercules, CA).

### Western Blot Assays

Western blot was performed as previously described [17]. In brief, blocked membranes were incubated with anti-Beclin1, anti-LC3B (Cell Signaling Technology, Beverly, MA), anti-S100A7, anti-Mfn1, anti-Mfn2, anti-β-actin (Santa Cruz), or anti-DRP1 (BD Biosciences, San Jose, CA) at a dilution of 1∶1,000 or 1∶2,000. Immunoreactive bands were visualized using a chemiluminescent ECL detection kit (Pierce, Rockford, IL).

### S100A7 Knockdown by Transient Transfection in HaCaT Cells

Three siRNA nucleotides targeting S100A7 and designed by GenePharma (Shanghai, China) were initially assayed. The one displaying the maximum S100A7 knockdown in HaCaT cells 48 h after transfection was subsequently used (sense: 5′-GCCGAUGUCUUUGAGAAAATT-3′, antisense: 5′-UUUUCUCAAAGACAUCGGCTT-3′). HaCaT cells were transfected with the S100A7-siRNA or NC-siRNA using lipofectamine 2000 (Invitrogen), according to the manufacturer’s instructions. Stretch experiments were carried out on cells 48 or 72 h post-transfection.

### Statistical Analysis

Data are expressed as the means ± SEM from at least three independent experiments. Statistical significance was calculated by one-way analysis of variance (ANOVA) and P value for a multiple comparison test using GraphPad Prism 5 statistical software, with *P* values <0.05 considered to be significant.

## Results

### Characterization of S100A7 Overexpression in HaCaT Cells

**Figure 1:**
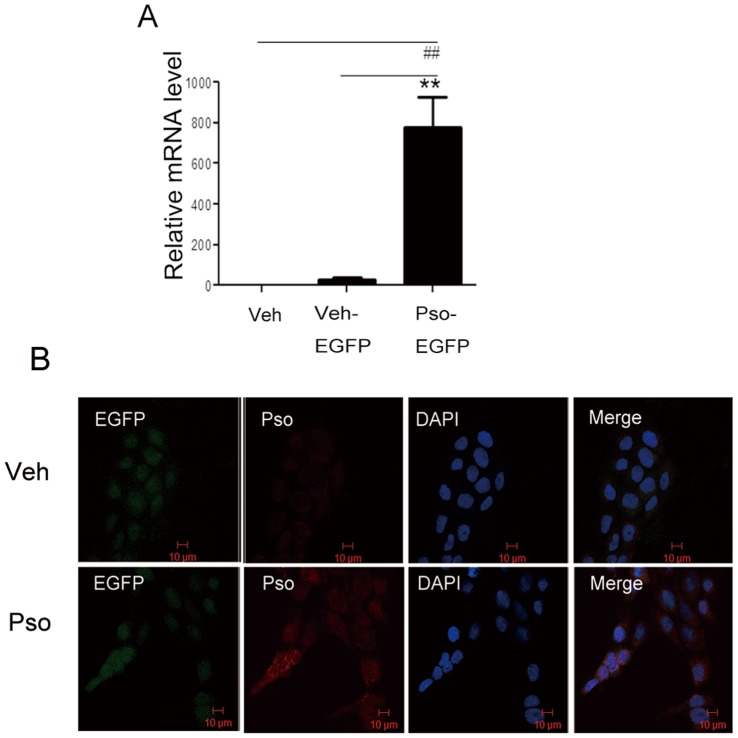
Characterization of S100A7 overexpression in HaCaT cells. (**A**) S100A7 mRNA levels in HaCaT cells overexpressing S100A7. S100A7 mRNA is normalized to β-actin. The data represent the mean ± SEM of three independent experiments. Statistical significance (**P<0.01, ^##^ *P*<0.01). (**B**) Immunofluorescence analysis of S100A7 overexpression. Cells were stained for EGFP (green) and anti-S100A7 (red); the nuclei were stained with DAPI (blue). Veh, vehicle; Veh-EGFP, vehicle-EGFP; pso-EGFP, S100A7-EGFP.

To investigate the mechanism of S100A7, we generated HaCaT cells constitutively expressing S100A7 (S100A7-EGFP cells) or EGFP alone (vehicle-EGFP cells). As shown in Figure 1A, after stable transfection, the expression of S100A7 mRNA was more abundant in the S100A7-EGFP HaCaT cells than in non-transfected HaCaT cells or vehicle-EGFP cells, as determined by quantitative PCR (Figure 1A). Consistent with S100A7 mRNA overexpression, immunofluorescence staining (red) showed that the S100A7-EGFP HaCaT cells strongly expressed S100A7 compared with the vehicle-EGFP cells (Figure 1B).

### S100A7 Up-regulated the Expression of Mitochondrial Biogenesis Genes

**Figure 2:**
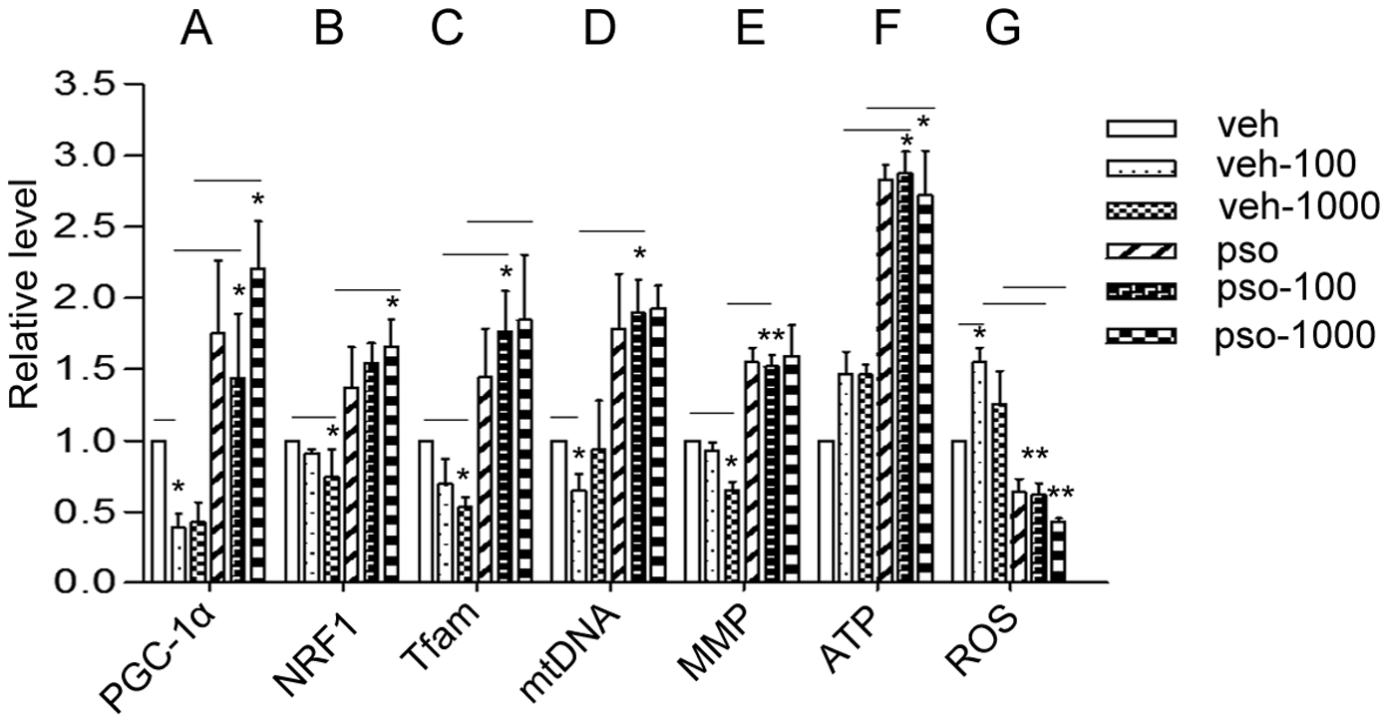
Effects of S100A7 overexpression on the expression of mitochondrial biogenesis and function. S100A7-EGFP and vehicle-EGFP HaCaT cells were exposed to lipopolysaccharide (LPS) (100 or 1000 ng/ml, 24 h). (**A**, **B**, **C**) The mRNA levels of PPAR-coactivator-1alpha (PGC-1α), nuclear respiratory factor-1 (NRF1) and transcription factor A (Tfam) were analyzed by quantitative RT-PCR. (**D**, **E**, **F**, **G**) Relative mitochondrial DNA contents (mtDNA), mitochondrial membrane potential (MMP), intracellular intracellular adenosine 5′-triphosphate (ATP) level and reactive oxygen species (ROS) generation were measured respectively. Values are means ± SEM; *Statistical significance (*P<0.05, **P<0.01). veh, vehicle; veh-100 or veh-1000, vehicle with 100 or 1000 ng/ml LPS treatment; pso, S100A7; pso-100 or pso-1000, S100A7-EGFP HaCaT cells with 100 or 1000 ng/ml LPS treatment.

PGC-1α is a co-activator that promotes mitochondrial biogenesis. PGC-1α mRNA expression was strongly suppressed in HaCaT cells after cells were challenged with 100 or 1000 ng/ml LPS for 24 h, while S100A7 overexpression prevented the LPS-induced suppression of PGC-1α mRNA expression (Figure 2A).

NRF1 is a key nuclear-encoded transcription factor that regulates the expression of a number of nuclear-encoded genes, including mitochondrial Tfam, which are important for mitochondrial function. Tfam is responsible for stability, maintenance and transcriptional control of mitochondrial DNA (mtDNA). Translocation of Tfam to the mitochondria is important for the initiation of mtDNA transcription and replication. NRF1 induces the expression of Tfam [22], [23]. Therefore, the relative NRF1 and Tfam mRNA levels were examined by qRT-PCR. Treatment with 100 and 1000 ng/ml LPS for 24 h resulted in decreased mRNA expression of NRF1 and Tfam, similar to the LPS-induced suppression of PGC-1α expression. However, S100A7 overexpression significantly up-regulated NRF1 and Tfam in HaCaT cells with or without LPS challenge (Figure 2B and 2C).

### S100A7 Overexpression Increased mtDNA Content

Tfam regulates the expression of various nuclear genes encoding mitochondrial proteins that regulate mtDNA transcription and replication. The level of Tfam expression is proportional to that of mtDNA [22], [23]. Because S100A7 stimulated the mRNA expression of Tfam, we hypothesized that the mtDNA copy number might also be increased. The mtDNA content was quantified by real-time PCR and measured as the ratio of D-loop to 18s rRNA levels. As shown in Figure 2D, treatment with 100 and 1000 ng/ml LPS for 24 h resulted in a decrease in the ratio of mitochondrial D-loop/18s rRNA. The overexpression of S100A7 resulted in a significant increase in mtDNA copy number (Figure 2D).

### S100A7 Protected Mitochondrial Function

MMP is an important index of mitochondrial function, which is closely related to ATP production. An increase in mitochondrial biogenesis should be accompanied by increased mitochondrial function [22], [23]. We examined MMP, ATP and ROS generation and found that exposure to 100 and 1000 ng/ml LPS for 24 h caused a decrease in MMP levels. A significant increase MMP was observed in S100A7-EGFP HaCaT cells than in vehicle-EGFP cells (Figure 2E). S100A7 overexpression effectively increased ATP production when compared with vehicle-HaCaT cells (Figure 2F). Treatment with 100 or 1000 ng/ml LPS for 24 h resulted in a significant increase in ROS production in HaCaT cells while S100A7 overexpression significantly inhibited the LPS-induced ROS production (Figure 2G).

**Figure 3:**
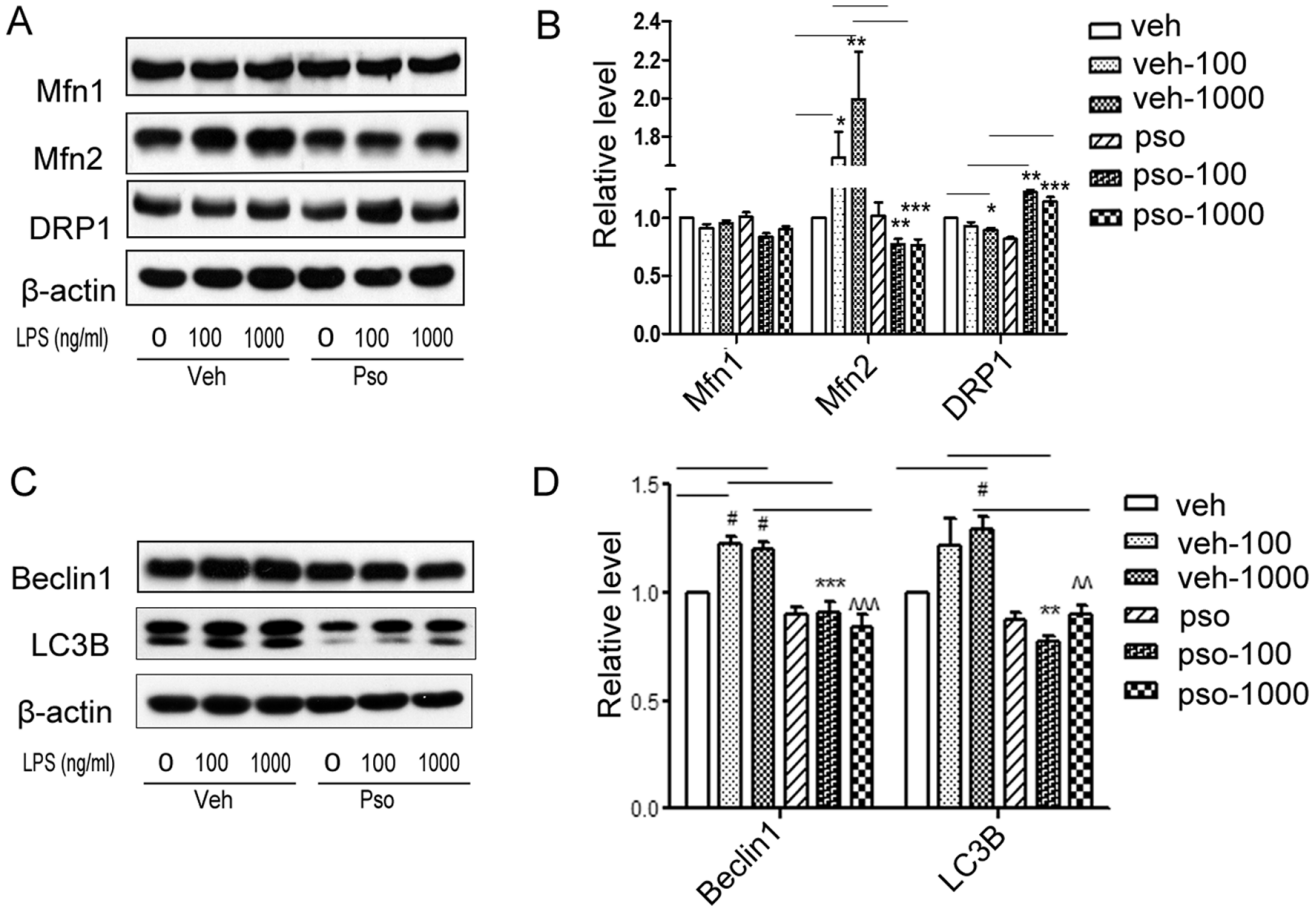
Effects of S100A7 overexpression on mitochondrial dynamics and autophagy activation. S100A7-HaCaT cells were treated for 24 h with LPS at 100 and 1000 ng/ml. (**A**, **B**) The mitochondrial dynamics-related proteins Mfn1, Mfn2 and DRP1 and (**C**, **D**) the autophagy-related proteins Beclin1 and LC3B were detected by Western blot (**A**, **C** western blot images; **B**, **D** statistical results). Values are means ± SEM. Statistical significance is indicated (*P<0.05, **P<0.01, ***P<0.001, ^#^ *P*<0.05, ∧∧*P*<0.01, ∧∧∧*P*<0.001). veh, vehicle; veh-100 or veh-1000, vehicle with 100 or 1000 ng/ml LPS treatment; pso, S100A7; pso-100 or pso-1000, S100A7-EGFP HaCaT cells with 100 or 1000 ng/ml LPS treatment.

Mitochondrial homeostasis is also regulated by mitochondrial fusion and fission which result in a continuous remodeling of the mitochondrial network [24]. In our study, we found that LPS (100 or 1000 ng/ml, 24 h) treatment led to an increase in the mitochondrial fusion-related protein, Mfn2, and significantly decreased the fission-related protein, DRP1. S100A7 overexpression significantly decreased the expression of Mfn2 and increased the expression of DRP1, without affecting Mfn1, compared to vehicle-EGFP cells challenged with LPS (100 or 1000 ng/ml, 24 h) (Figure 3A and 3B). Thus, we confirmed that S100A7 could regulate mitochondrial homeostasis and could stimulate mitochondrial biogenesis in HaCaT cells.

### S100A7 Down-regulated the Expression of Autophagy-related Proteins

Western blot analysis showed that the autophagy-related proteins Beclin1 and LC3B were highly induced by LPS (100 or 1000 ng/ml, 24 h) in vehicle-EGFP HaCaT cells and that S100A7 overexpression significantly inhibited the increase in Beclin1 and LC3B compared with vehicle-EGFP cells induced by LPS (Figure 3C and 3D).

### S100A7 Increased IL-6 and IL-8 Production in Immortalized Keratinocytes

**Figure 4:**
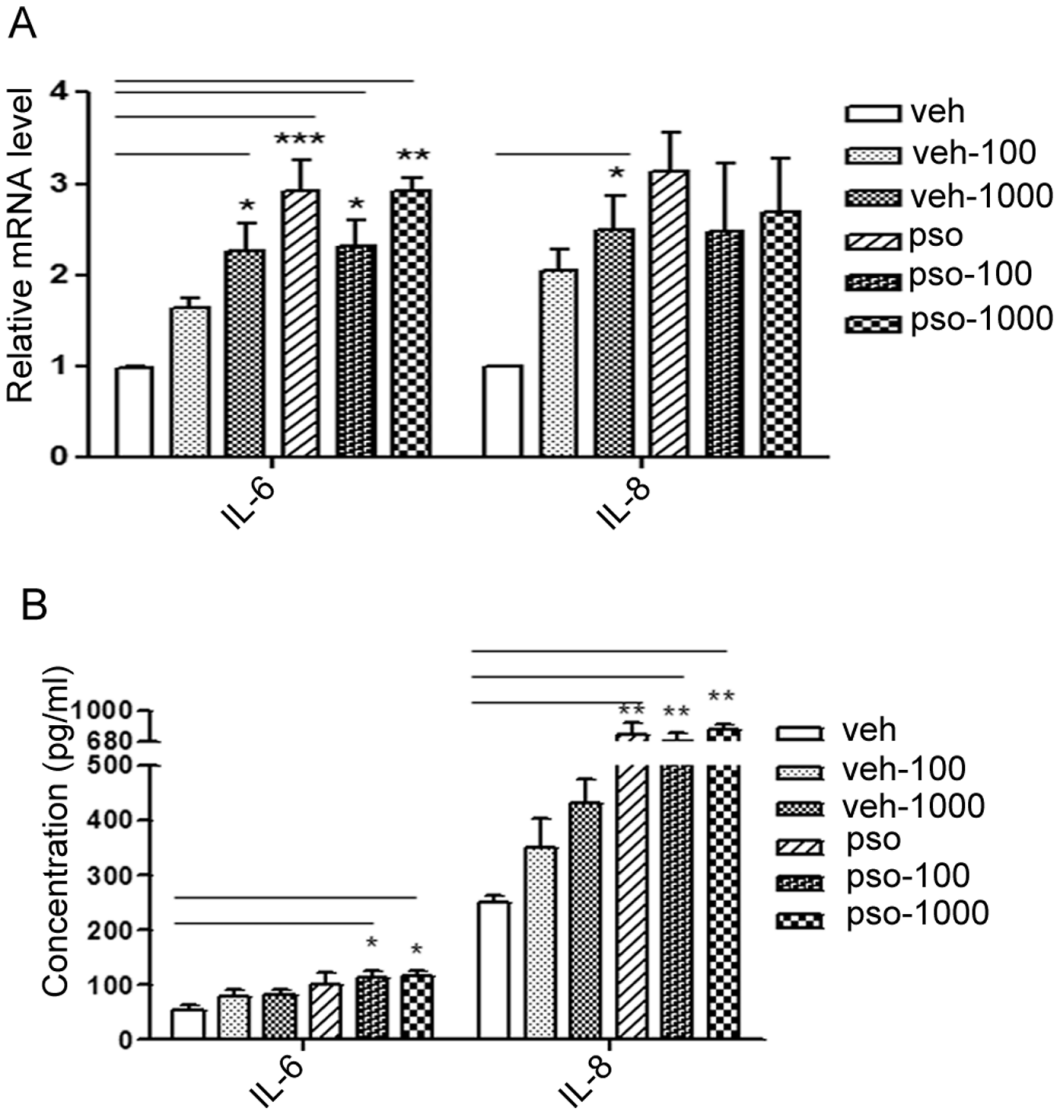
Effects of S100A7 overexpression on the LPS-induced mRNA and protein expression of IL-6 and IL-8. (**A**) IL-6 and IL-8 mRNA levels and (**B**) protein levels were assayed after stable HaCaT cells (S100A7-EGFP or vehicle-EGFP) were exposed to LPS (100 or 1000 ng/ml, 24 h). The data represent the means ± SEM of three independent experiments. Statistical significance is indicated (*P<0.05, **P<0.01, ***P<0.001). veh, vehicle; veh-100 or veh-1000, vehicle with 100 or 1000 ng/ml LPS treatment; pso, S100A7; pso-100 or pso-1000, S100A7-EGFP HaCaT cells with 100 or 1000 ng/ml LPS treatment.

After treating S100A7-EGFP cells and vehicle-EGFP HaCaT cells with 100 and 1000 ng/ml LPS for 24 h, mRNA levels of IL-6, IL-8, TNF-α, IL-1α and IL-1β were measured by quantitative RT-PCR and protein levels were measured by ELISA. LPS increased production of IL-6 and IL-8, which are related to inflammatory cutaneous processes, compared with the vehicle-EGFP cells. S100A7 overexpression significantly increased IL-6 and IL-8 production with or without LPS (100 or 1000 ng/ml, 24 h) compared to the vehicle-EGFP group (Figure 4A and 4B). Stimulation with LPS (100 or 1000 ng/ml, 24 h) in HaCaT cells overexpressing S100A7 did not significantly increase TNF-α, IL-1α or IL-1β mRNA or protein levels (Data not shown).

### Knockdown of S100A7 Decreased MMP and Disrupted Mitochondrial Homeostasis

**Figure 5:**
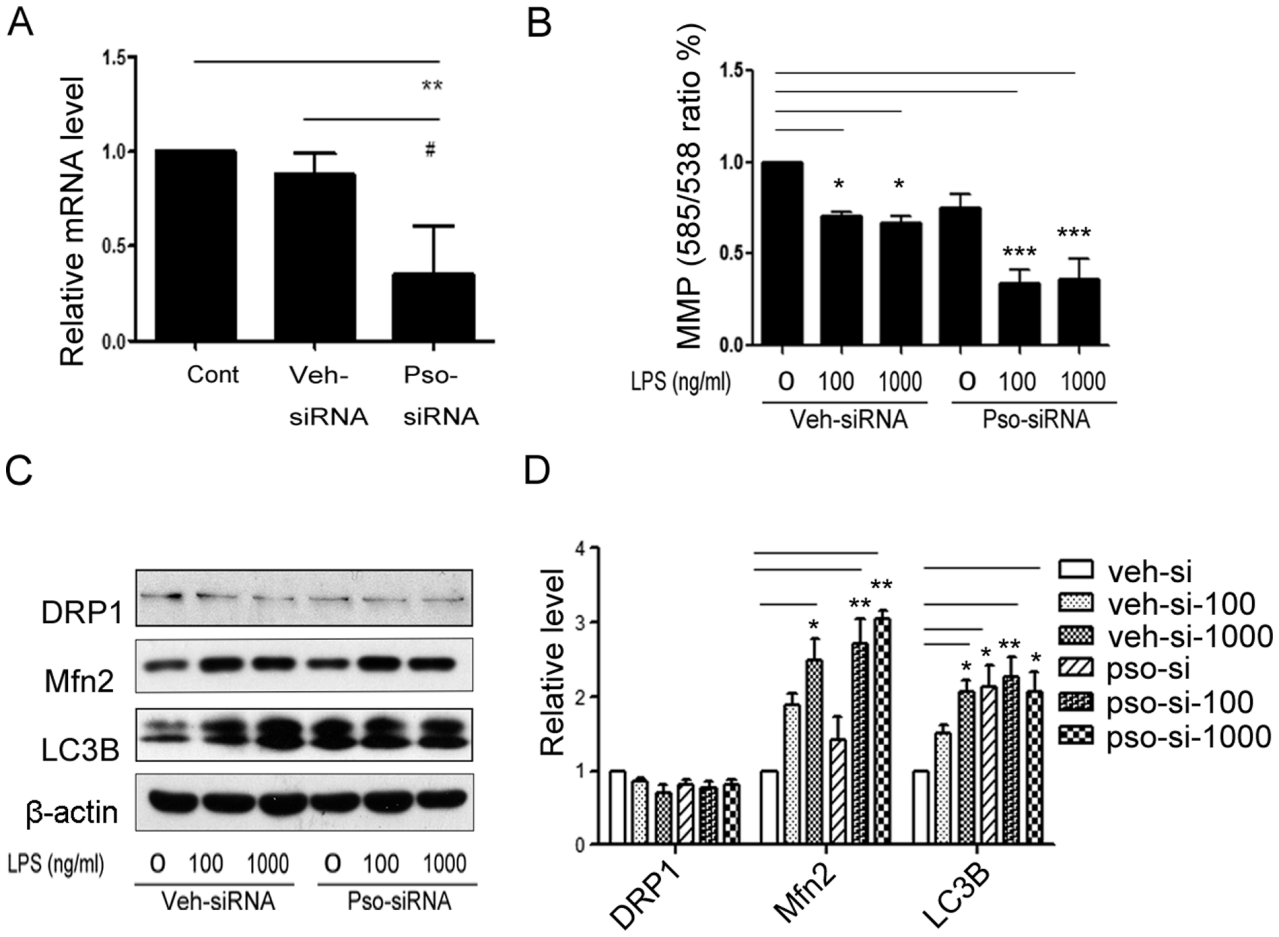
Effects of S100A7 knockdown on MMP and mitochondrial homeostasis. (**A**) Expression of S100A7 mRNA after siRNA transfection. HaCaT cells were transfected with S100A7 siRNA using lipofectamin 2000 for 48 h. Total RNA was extracted and subjected to quantitative RT-PCR. The amount of S100A7 mRNA relative to β-actin is shown. (**B**) S100A7-siRNA HaCaT cells were treated for 24 h with LPS at 100 and 1000 ng/ml. The MMPs were assayed. (**C**, **D**) The mitochondrial dynamics-related proteins DRP1 and Mfn2 and the autophagy-related protein LC3B were detected by Western blot (**C**, Western blot images; **D**, statistical results). Values are means ± SEM. Statistical significance is indicated (^#^ *P*<0.05, *P<0.05, **P<0.01, ***P<0.001). cont, control; veh-si, vehicle-siRNA; veh-si-100 or veh-si-1000, vehicle-siRNA cells with 100 or 1000 ng/ml LPS treatment; pso-si, S100A7-siRNA; pso-si-100 or pso-si-1000, S100A7-siRNA HaCaT cells with 100 or 1000 ng/ml LPS treatment.

**Figure 6:**
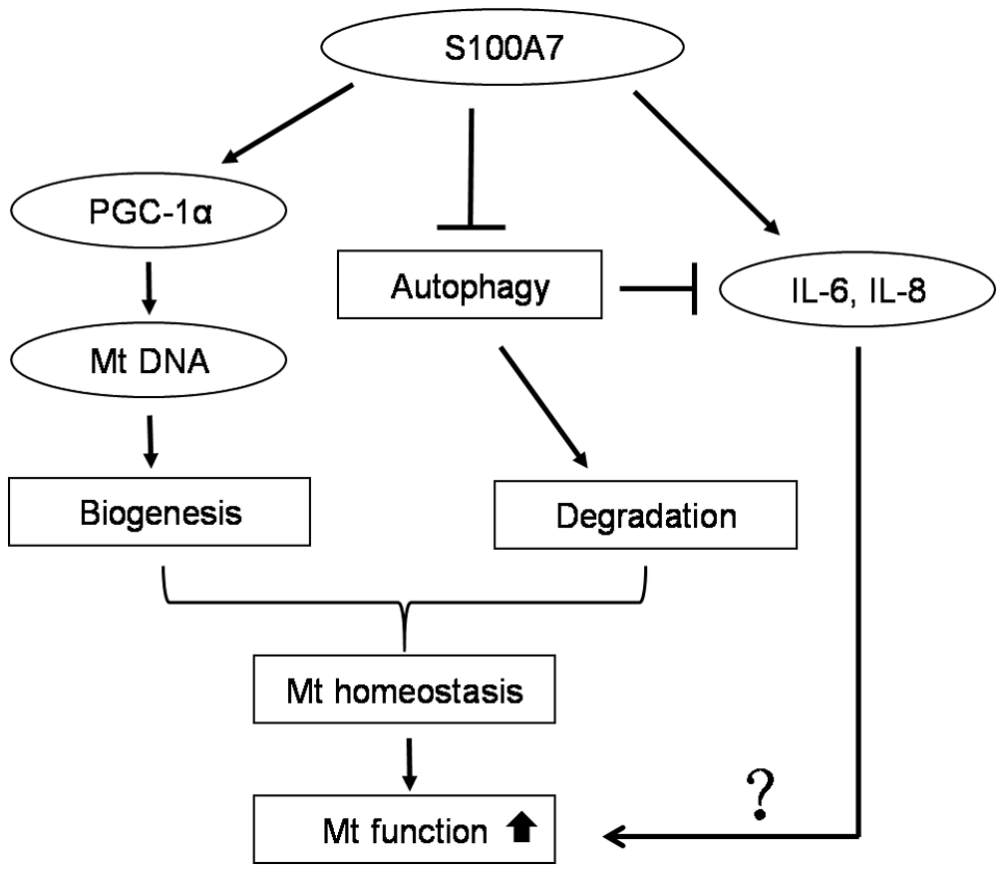
Schematic illustration of the possible mechanisms of S100A7 protection against LPS-induced mitochondrial dysfunction. S100A7 stimulates mitochondrial biogenesis by up-regulating PGC-1α signaling pathway. S100A7 down-regulates autophagy but promotes inflammation. The mechanism of S100A7 attenuates HaCaT cells damage induced by LPS is mediated by mitochondrial homeostasis through biogenesis and degradation to increase mitochondrial function. Mt: mitochondrion.

To better understand how influential S100A7 is on mitochondrial function and biogenesis in HaCaT cells, we used siRNA to knockdown S100A7 in HaCaT cells. As shown in Figure 5A, S100A7 mRNA was decreased in S100A7-siRNA HaCaT cells at the 48 h time point compared with the negative control (vehicle-siRNA) and the non-transfected control groups (Figure 5A). Next, we determined the effects of S100A7 knockdown on the mitochondrial membrane potential. The addition of LPS (100 or 1000 ng/ml, 24 h) to S100A7 knockdown cells led to a decrease in the expression of MMP compared with the vehicle-siRNA group (Figure 5B). Treatment of vehicle-siRNA HaCaT cells and S100A7-siRNA cells with LPS (100 or 1000 ng/ml, 24 h) led to a significant increase in Mfn2 expression. But the addition of LPS to vehicle-siRNA cells and to S100A7-siRNA cells showed no significant decreases in DRP1 expression. Meanwhile, silencing S100A7 led to significant increases in the expression of the autophagy-related protein, LC3B (Figure 5C and 5D). The effect of S100A7 knockdown is equivalent to the exogenous addition of LPS (100 or 1000 ng/ml, 24 h) in HaCaT cells. Our results may be summarized in a mechanistic model of how S100A7 attenuates HaCaT cells damage stimulated with LPS by activating mitochondrial biogenesis and promoting mitochondrial function (Figure 6).

## Discussion

Epithelial surfaces harbor an abundance of microbes which surprisingly rarely lead to infections. The stratum corneum of the skin acts as a physical barrier that separates the body from the environment. S100A7 was described to have antimicrobial activity and to play an important role in surface defense [25]. It is expressed in normal, human differentiated keratinocytes and it is highly up-regulated in skin diseases, including skin inflammation and invasive skin cancers [26].

Mitochondria play critical roles in the life and death of cells. Mitochondrial dynamics maintain normal mitochondrial function by degrading damaged mitochondria and generating new mitochondria. In the present study, we showed that S100A7 stimulated the protein expression of PGC-1α, the central factor for mitochondrial biogenesis and the mRNA levels of its downstream targets, NRF1 and Tfam (Figure 2A, 2B and 2C). We used mtDNA copy number to evaluate mitochondrial biogenesis. S100A7 increased the quantity of mtDNA and, consequently, the number of mitochondria increased (Figure 2D). We also found that S100A7 maintained mitochondrial homeostasis through regulated mitochondrial fusion and fission in LPS-induced HaCaT cells. As shown in Figure 3, S100A7 overexpression decreased Mfn2 and increased DRP1 significantly (Figure 3A and 3B). When we knocked down S100A7 in LPS-induced HaCaT cells, expression of the fusion protein Mfn2 significantly increased (Figure 5C and 5D).

The mitochondrion is vital to cellular energy and metabolism, being the site of the generation of most ATP. Additionally, mitochondria are the main sources of endogenous ROS. Various stress conditions, including increased metabolic rates, hypoxia or membrane damage all markedly induce mitochondrial ROS production leading to mitochondrial dysfunction [27]. We found that increasing mtDNA copy numbers and mitochondrial biogenesis were accompanied by the enhancement of mitochondrial function, including an increase in ATP levels and a decrease in ROS generation (Figure 2F and 2G).

Autophagy is a homeostatic and dynamic process for the intracellular recycling of cytoplasm, proteins, lipids, and organelles [28]. We detected the involvement of autophagy by examining Beclin1 and LC3B. As shown in Figure 3C and 3D, LPS caused significant increases in the expression of Beclin1 and LC3B in HaCaT cells, suggesting that autophagy is greatly enhanced as a defense mechanism for the clearing of damaged mitochondria and unnecessary proteins. S100A7 overexpression effectively prevented the increases in Beclin1 and LC3B, suggesting that no significant accumulation of damaged mitochondria and unnecessary proteins had occurred with or without LPS (Figure 3C and 3D). S100A7 knockdown significantly increased the expression of the autophagy-related protein LC3B (Figure 5C and 5D).

In addition to their antimicrobial properties [25] [29], previous studies have shown that released S100A7 can function as a chemotactic agent and can activate various inflammatory cells, including neutrophils. It also regulates immunomodulatory activities in keratinocytes, including the enhancement of cytokine production, chemotaxis, cell proliferation and wound healing [30], [31]. Moreover, TNF-α, butyrate, VD3, EGF, PMA, flagellin and IL-1β significantly enhanced the expression of S100A7 in keratinocytes [32]. S100A7 was identified as chemotactic for granulocytes, monocytes and lymphocytes. S100A7 provoked an inflammatory response that was dose- and time-dependent and was mediated by RAGE, indicating that the identified S100A7 interaction with RAGE may provide a novel therapeutic target for inflammation [33].

Based on these previous studies, we concluded that some cytokines, such as IL-8, could induce the expression of S100A7 and S100A7 could activate the production of IL-6 and IL-8. The current study shows that S100A7 overexpression enhances the secretion of IL-6 and IL-8. The effect is similar to the exogenous addition of LPS in HaCaT cells (Figure 4A and 4B). Consistent with the report that autophagy could control inflammation to limit the production of the inflammatory cytokines [34], S100A7 down-regulates autophagy but promotes inflammatory cytokines secretion. The mechanism of S100A7 attenuates mitochondrial dysfunction is mediated by mitochondrial homeostasis through biogenesis and degradation in HaCaT cells (Figure 6). IL-6 induces a PI 3-kinase and NO-dependent protection and preserves mitochondrial function in cardiomyocytes [35]. IL-6 protects against hyperoxia-induced lung mitochondrial damage via Bcl-2-induced bak interactions with mitofusions [36]. Thus, we speculate that IL-6 and IL-8 may interact with specific receptors to affect mitochondrial function. Additional studies are necessary to confirm the mechanism of IL-6 and IL-8 in influencing mitochondrial function in HaCaT cells induced by LPS.

The MAPK pathway has been shown to be involved in a large variety of cellular activities, ranging from cell survival and proliferation to the expression of pro-inflammatory cytokines and chemokines [37], [38]. TNF-α-induced S100A7 expression is regulated by a p38-MAPK and ERK1/2-dependent mechanism in cultured human keratinocytes [39]. IL-36 cytokines enhance the production of S100A7 in human keratinocytes through the activation of MAPKs and NF-κB [40]. S100A7 activates neutrophils to produce IL-6, IL-8, TNF-α, macrophage inflammatory protein-1α (MIP-1α), MIP-1β and MIP-3α through the phosphorylation of p38 MAPK and ERK [41]. STAT3 and NF-κB regulate S100A7 expression in human bronchial epithelial cells [42]. S100A7 and epidermal fatty acid binding protein (E-FABP), a potential target of S100A7, were shown to interact in cultured normal human keratinocytes [43]. Therefore, it will be necessary to investigate MAPK and NF-κB activation along with specific receptors which interact with S100A7 to elucidate the mechanism by which S100A7 might stimulate keratinocytes.

Taken together, our results show that S100A7 is a promoter of mitochondrial biogenesis and function and it increases IL-6 and IL-8 secretion in HaCaT cells both when challenged by LPS or not challenged. These data suggest that S100A7 may potentially have therapeutic applications in treating inflammatory skin diseases. Further study of the role of S100A7 in skin inflammation is warranted and will be necessary to gain a clear understanding of the underlying mechanisms by which it is involved in these processes.
